# Uremic pruritus: prevalence, determinants, and its impact on health-related quality of life and sleep in Indian patients undergoing hemodialysis

**DOI:** 10.1007/s11845-023-03393-8

**Published:** 2023-05-12

**Authors:** Deeksha Shetty, Ajith M. Nayak, Divya Datta, Mohan V. Bhojaraja, Shankar Prasad Nagaraju, Attur Ravindra Prabhu, Dharshan Rangaswamy, Indu Ramachandra Rao, Srinivas Vinayak Shenoy, Dhruv Joshi

**Affiliations:** 1 Renal Replacement Therapy and Dialysis Technology, Manipal College of Health Professions, Manipal Academy of Higher Education, Manipal, 576104 Karnataka India; 2 Department of Nephrology, Kasturba Medical College, Manipal, Manipal Academy of Higher Education, Manipal, 576104 Karnataka India

**Keywords:** End-stage kidney disease, Hemodialysis, Quality of life, Sleep, Uremic pruritus

## Abstract

**Background:**

Uremic pruritus has an impact on the quality of life and sleep of hemodialysis patients, but the majority of cases go unreported and untreated unless severe, due to a lack of awareness. The purpose of this study is to determine the prevalence, associated factors, and impact on health-related quality of life (HR-QOL) and sleep in hemodialysis patients.

**Methodology:**

A single-center observational study of 3 months wherein 120 adults on maintenance hemodialysis were included. Baseline characteristics, dialysis-related factors, and lab parameters influencing uremic pruritus were recorded. Those with uremic pruritus completed “12-item pruritus severity scale (12-PSS)”, “SKINDEX10”, and “Itch-MOS” questionnaires to evaluate severity, impact on HR-QOL, and sleep respectively.

**Results:**

Sixty seven over one hundred twenty (55.83%) patients had pruritus and majority were mild (40.83%) as per 12-PSS. Those with pruritus (n=67) had a mean age of 56.5±11.3 years, most were males (82%), chronic glomerulonephritis (29.1%) was the commonest cause of end-stage kidney disease, 3 active smokers, and 4 seropositive. 65(97%) patients were on twice-weekly dialysis, 36/67 had <5 years’ dialysis vintage and acceptable adequacy. There was no significant association between uremic pruritus and dialysis-related/laboratory parameters. Patients with uremic pruritus demonstrated significantly worse “HR-QOL” (p<0.001) on the “SKINDEX-10”, and patients' “Itch-MOS” scores demonstrated a significant decline in sleep quality with increasing pruritus severity (p<0.001).

**Conclusion:**

The majority of patients on maintenance hemodialysis experience uremic pruritus. None of the clinical characteristics, dialysis-related factors, and laboratory parameters affected uremic pruritus. Uremic pruritus patients had the worst HR-QOL & their sleep quality significantly declined as pruritus severity escalated.

**Trial registration number and date of registration:**

Study approval was obtained from Institutional Research Committee and Institutional Ethical Committee (IEC 642/2021). Clinical Trial Registry of India (CTRI) registration (CTRI/2022/01/039143) was also obtained.

## Introduction

Pruritus is a distressing symptom that is common in patients with chronic kidney disease (CKD) and end-stage kidney disease (ESKD) on dialysis and has a negative impact on these patients' quality of life (QOL). The pruritus caused by CKD known as "uremic pruritus" is often used interchangeably with “CKD-associated pruritus (CKD-aP)” [1].

The prevalence of CKD-aP has varied greatly between studies. It has been reported that it can range from 20 to 90%. In “DOPPS I” 45% of patients had moderate to severe pruritus, while in “DOPPS II” the prevalence was 42%. In “DOPPS phase V” (2012–2015), the number of patients with pruritus decreased from 28 to 18% [2–5]. In a meta-analysis of 42 cross-sectional studies, the prevalence of pruritus in HD patients was similar to that in PD patients (55% vs. 56%) [6].

The timing and frequency of the itching can vary, and the severity of pruritus can range from mild to severe. The following criteria must be met in order to diagnose uremic pruritus: more than or equal to three episodes of itching during a period of two weeks, with the symptom appearing a few times per day, lasting at least a few minutes, and bothering the patient; and the appearance of an itch in a regular pattern during a period of six months, but less frequently [7].

Uncertainty exists regarding the pathogenesis. To explain it, numerous theories have been put forth in numerous studies such as immune dysregulation, xerosis, hyperparathyroidism, uremic toxin accumulation, neural dysfunction, histamine mechanism, opioid mechanism, and proteinase-activated receptors (PARs)2 pathway. Risk factors associated are older age, male patients, hypertension, diabetes, smoking within the past year, the presence of hepatitis B or C antibodies, low hemoglobin levels, high phosphate, calcium, PTH levels and negatively correlated with Kt/V and serum albumin levels [8].

Patients experience significant distress as a result of uremic pruritus, which has a major impact on their QOL, and those with lower QOL were found to have a higher mortality rate. It can have an impact on their sleep and social functioning as well by keeping them awake at night, causing them to feel drowsy during the day and, as a result, not get enough sleep, affecting their daily activities [9].

Despite advancements in the care of hemodialysis patients, controlling pruritus remains one of the most challenging clinical manifestations for nephrologists to manage. As there is limited knowledge on the prevalence of pruritus, its association with clinical, dialysis-related, and metabolic parameters, its effect on “HR-QOL” and sleep in the Indian hemodialysis population, in this study we analysed the same at a tertiary care hospital.

## Methodology

### Study sample

A cross-sectional, single-center, time-bound observational study of 3 months was conducted in 2022 at a tertiary care hospital on 120 eligible participants who were enrolled using the convenience sampling technique. Study approval was obtained from Institutional Research Committee and Institutional Ethical Committee (IEC 642/2021). Clinical Trial Registry of India (CTRI) registration (CTRI/2022/01/039143) was done and informed consent was taken from all the participants. All patients aged > 18 years and on regular hemodialysis for > 3 months were analysed and those on peritoneal dialysis, skin diseases other than uremic pruritus, and those with acute kidney injury were excluded.

### Data collection

The demographic details & medical histories such as age, gender, etiology of ESKD, smoking status, seropositivity, and whether on anti-pruritic treatment were recorded. Dialysis-related parameters included were dialysis vintage, duration of each session, frequency of dialysis, and dialysis adequacy (Kt/v). Laboratory parameters affecting pruritus such as hemoglobin, albumin, calcium, phosphorus, and parathyroid hormone levels were checked in all patients in the study.

The 12-point Pruritus Severity Scale *(12 PSS)* was used to determine uremic pruritus prevalence and severity. The impact on HR-QOL was analysed based on symptoms, emotional and functional domains using the standard SKINDEX-10 questionnaire, and the influence of uremic pruritus on sleep quality & quantity was based on the standard Itch-MOS questionnaire.

### Outcome measures

The primary outcome was to study the prevalence and intensity of uremic pruritus in patients on regular hemodialysis. Secondary outcomes were to study clinical characteristics, dialysis-related factors, and laboratory parameters influencing uremic pruritus as well as to analyse its impact on HR-QOL and sleep.

### Statistical analysis

Categorical data were expressed as frequency and relative frequency; continuous data were expressed as mean and standard deviation. Categorical data were compared using the "Chi-square^2^ test" and “Analysis of variance (ANOVA)” test for comparing quantitative data. “Post hoc analysis” was used to analyse specific differences between the means of the 3 groups if the “ANOVA test” was significant. Statistical significance is defined as a p-value < 0.05. For analysis, the “Statistical Package for the Social Science (SPSS) 20.0” was used.

## Results

### Baseline characteristics

The study included 120 people who were on maintenance hemodialysis and met the inclusion criteria. 67/120 (55.8%) patients had pruritus in our study. Uremic pruritus patients (n = 67; 55.8%) had a mean age of 56.5 ± 11.3 years, most were males (n = 55;82%). Among etiology for ESKD, chronic glomerulonephritis (CGN) (n = 35; 29.1%) was the commonest, 16 had diabetic kidney disease, 9 had chronic tubulointerstitial nephritis (CTIN), 9 had hypertensive nephrosclerosis (HS) and 1 had autosomal dominant polycystic kidney disease (ADPKD). 3 were active smokers, and 4 were seropositive (3 “hepatitis B” positive and 1 “hepatitis C” positive). The majority of these patients were treated with oral antihistamines (n = 45; 67.2%), the remaining were managed with topical applications (n = 17; 25.4%), and very few required oral pregabalin (n = 5; 7.4%) (Table 1). Most of these patients had no obvious skin lesions (n = 60; 89.5%), however, 4 of them had crusts with papules and 3 had erosions with ulcerations.

**Table 1:** Baseline characteristics

**Baseline Characteristics**	**Non-pruritus** **(n = 53; 44%)**	**Pruritus** **(n = 67; 56%)**	**Total patients** **(n = 120)**
**Age (in years)**			
	52.3 ± 14.5	56.5 ± 11.3	
**Gender distribution**			
**Males**	39 (73.5%)	55 (82%)	94 (78.4%)
**Females**	14 (26.5%)	12 (18%)	26 (21.6%)
**Etiology of CKD**			
**DKD**	6 (11.3%)	16 (23.8%)	22 (18.3%)
**CGN**	11 (20.7%)	24 (35.8%)	35 (29.1%)
**CTIN**	18 (34%)	9 (13.4%)	27 (22.5%)
**HNS**	9 (17.1%)	9 (13.4%)	18 (15%)
**ADPKD**	3 (5.6%)	1 (1.6%)	4 (3.3%)
**Others**	6 (11.3%)	8 (12%)	14 (11.6%)
**Smokers**			
**Yes**	2 (3.8%)	3 (4.5%)	5 (4.1%)
**No**	51 (96.2%)	64 (95.5%)	115 (95.9%)
**Seropositive patients**			
**HBV**	None	3 (100%)	3 (2.5%)
**HCV**	None	1 (100%)	1 (0.8%)
**Medications/Therapies**			
**Topical applications**	-	17 (25.4%	-
**Oral antihistaminic**	-	45 (67.2%)	-
**Pregabalin**	-	5 (7.4%)	*-*

*DKD* Diabetic kidney disease, *CGN* chronic glomerulonephritis, *CTIN* chronic tubulointerstitial nephritis, *HNS* hypertensive nephrosclerosis, *ADPKD* autosomal dominant polycystic kidney disease, *HBV* hepatitis B virus, *HCV* hepatitis C virus

### Primary outcome

**Fig. 1:**
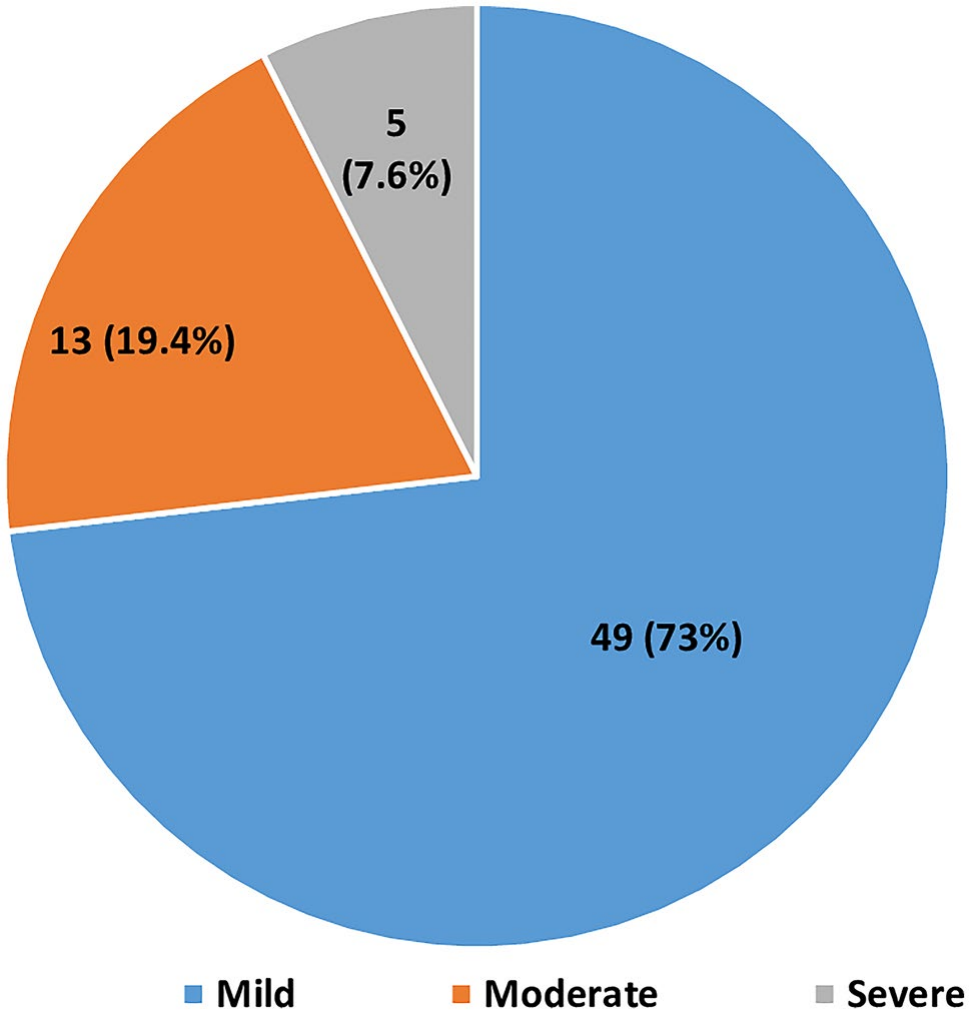
Prevalence of uremic pruritus based on the 12-item pruritus severity scale

In our study, uremic pruritus affected 55.8% of the participants (67/120 patients). Based on the 12-PSS scale they were further divided into three categories based on their severity: the majority was “Mild” (n = 49; 40.8%), “Moderate” (n = 13; 19.4%) & “Severe” (n = 5; 4.1%) (Fig. 1)*.*

### Secondary outcomes

#### Determinants of uremic pruritus

There was no significant association between 67 patients with uremic pruritus when compared with their clinical characteristics in terms of age, gender, etiology of ESKD, smoking, and seropositive status (Tables 1 and 2); 36/67 patients had dialysis vintage of < 5 years, 31/67 had a vintage of > 5 years, 60/67 and 2/67 patients were on twice and thrice weekly hemodialysis respectively. In terms of adequacy, the Kt/V in those with “mild, moderate, and severe” pruritus was 1.41 ± 0.28, 1.35 ± 0.22, and 1.47 ± 0.28 respectively which was acceptable. However, none of the analysed dialysis-related factors correlated significantly. Laboratory parameters such as hemoglobin, serum albumin, calcium, phosphorus, and parathormone had no association with the occurrence of uremic pruritus (Table 2)*.*

**Table 2:** Comparison of parameters with the severity of pruritus

**Characteristics**	**No Pruritus** **(n = 53; 44%)**	**Pruritus** **(n = 67; 56%)**			**p-value**
		**Mild** **(n = 49; 73%)**	**Moderate** **(n = 13; 19.4%)**	**Severe** **(n = 5; 7.6%)**	
**Age (in years)**	52.3 ± 14.5	54.3 ± 11.3	58.3 ± 15.6	57 ± 6.9	0.46
**Gender**					
**Males**	39(73.5%)	40(81.6%)	10 (76.9%)	5(100%)	0.49
**Females**	14 (26.5%)	9(18.4%)	3(23.1%)	None	
**Etiology of ESKD**					
**DKD**	6(11.3%)	11 (22.4%)	3 (23%)	2 (40%)	0.25
**CGN**	11 (20.7%)	17 (34.6%)	6 (46.2%)	1 (20%)	0.2
**CTIN**	18 (34%)	6 (12.3%)	2 (15.4%)	1 (20%)	0.06
**HNS**	9 (17.1%)	7 (14.3%)	2 (15.4%)	None	0.78
**ADPKD**	3 (5.6%)	None	None	1 (20%)	0.06
**Others**	6 (11.3%)	8 (16.4%)	None	None	0.33
**Seropositive**					
**HBV**	None	1 (2%)	None	2 (40%)	
**HCV**	None	1 (2%)	None	None	
**Smoking Status**					
**Smokers**	2 (3.8%)	3 (6.1%)	None	None	0.73
**Non Smokers**	51 (96.2%)	46 (93.9%)	13(100%)	5(100%)	
**Dialysis Vintage**					
**< 5 years**	30 (56.6%)	28 (57.1%)	6 (46.1%)	2 (40%)	0.79
**> 5 years**	23 (43.4%)	21 (42.9%)	7 (53.9%)	3 (60%)	
**Dialysis Adequacy**					
**Kt/v**	1.53 ± 0.38	1.41 ± 0.28	1.35 ± 0.22	1.47 ± 0.28	0.17
**Dialysis Frequency (per week)**					
**twice**	47 (88.6%)	48 (97.9%)	12 (92.3%)	5 (100%)	0.27
**thrice**	6 (11.4%)	1 (2.1%)	1 (7.7%)	None	
**Laboratory parameters**					
**Hemoglobin (g/dl)**	10.06 ± 1.51	9.65 ± 1.31	9.8 ± 1.9	9.12 ± 1.17	0.37
**Albumin (g/dl)**	4.03 ± 0.41	4 ± 0.35	4.32 ± 0.44	3.98 ± 0.34	0.06
**Calcium (mg/dL)**	8.66 ± 0.72	8.53 ± 0.78	8.07 ± 0.9	8.56 ± 0.62	0.11
**Phosphorus (mg/dL)**	5.09 ± 1.91	5.23 ± 1.85	1.62 ± 0.45	4.78 ± 1.33	0.58
**PTH (pg/mL)**	216(106,529)	340(154,529)	212(145,452)	150(290,505)	0.45
**SKINDEX-10**					
**Symptoms**	None	5 (2,7)	8 (6,10)	13 (12,16)	** < 0.001**
**Emotions**	None	4 (3,7)	9 (7,11)	11 (9,15)	** < 0.001**
**Functioning**	None	4 (2,8)	10 (4,12)	10 (6.5,21.5)	**0.01**
**itch-MOS score**					
**Total Score**	None	44.91 ± 8.03	41.23 ± 7.91	40.91 ± 7.32	0.67
**Sleep hours/day**	6.65 ± 1.31	6.67 ± 1.74	5.76 ± 1.69	3 ± 1.41	** < 0.001**

*DKD* Diabetic kidney disease, *CGN* chronic glomerulonephritis, *CTIN* chronic tubulointerstitial nephritis, *HNS* hypertensive nephrosclerosis, *ADPKD* autosomal dominant polycystic kidney disease, *HBV* hepatitis B virus, *HCV* hepatitis C virus

#### HR-QOL and sleep

With respect to symptoms (p 0.001), emotions (p 0.001), and functionality (p = 0.012), SKINDEX-10 showed significantly worse HR-QOL in patients with uremic pruritus (Table 2)*.*

**Fig. 2:**
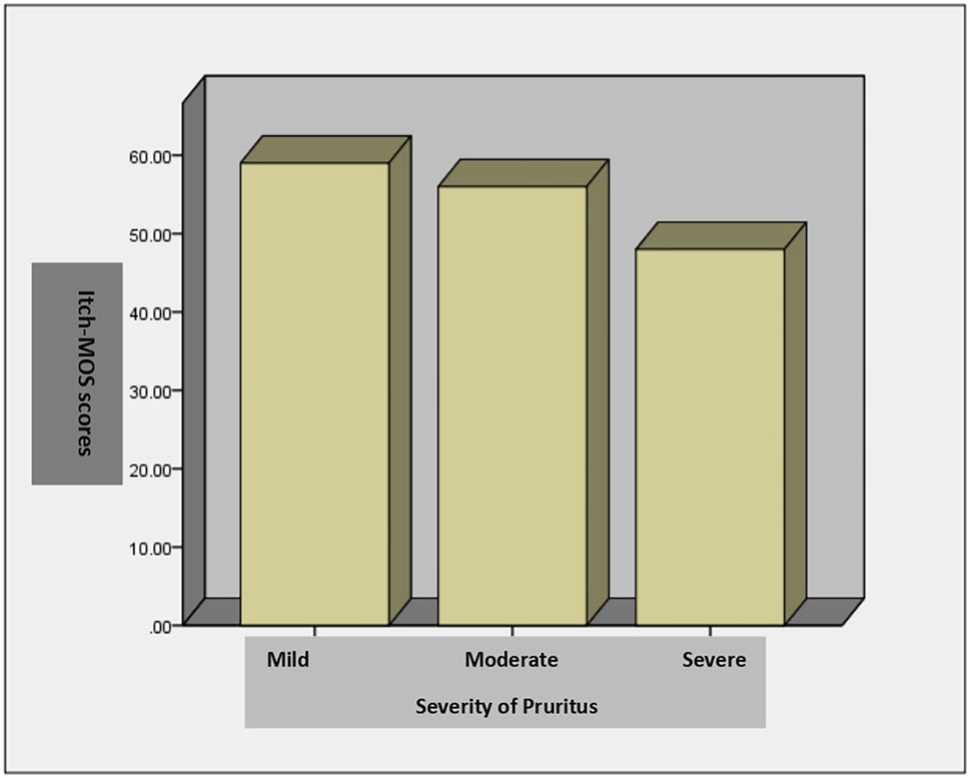
Comparison of Itch-MOS scores and severity of pruritus

**Fig. 3:**
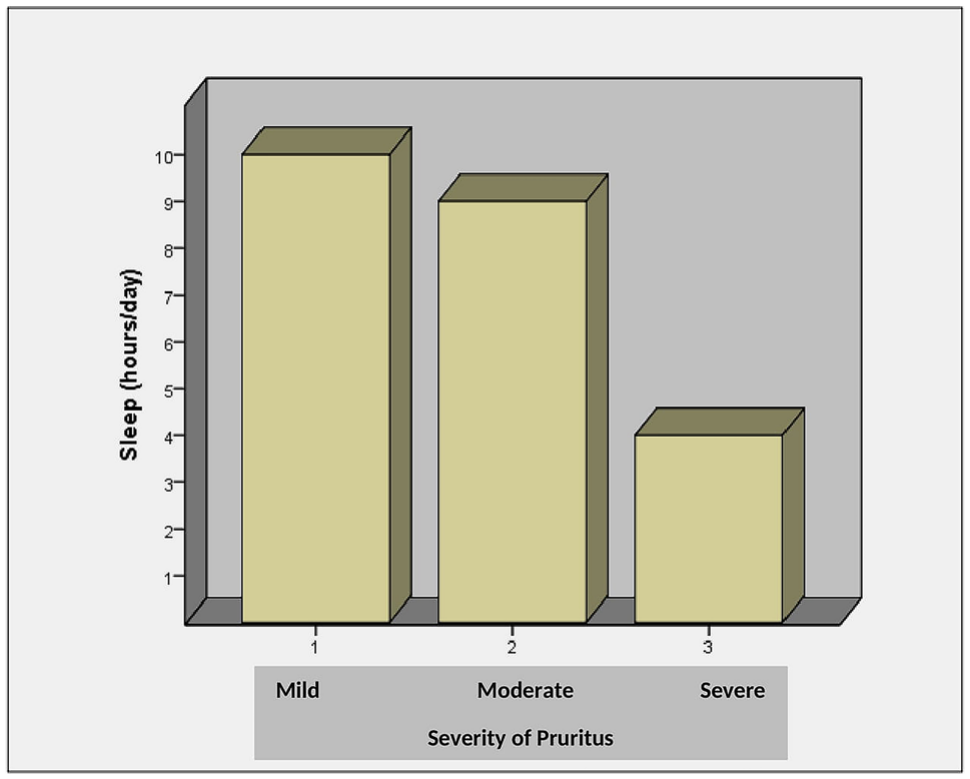
Comparison of sleep hours and severity of pruritus

The severity of pruritus was significantly correlated with the Itch-MOS score (p =  < 0.001) (Table 2) confirming that the quality of sleep reduced with the increasing severity of pruritus (Figs. 2 and 3)*.*

A “Post hoc analysis” was used to analyse specific differences between the means of these three groups of SKINDEX-10 which showed a statistically significant difference between the groups in terms of symptoms and emotions (Table 3)*.*

**Table 3:** Post hoc test-multiple comparison of SKINDEX-10 Score

**Symptoms**	p-value*
No-Moderate	**0.002**
No-Severe	** < 0.001**
Mild-Severe	**0.003**
**Emotions**	
No-Moderate	** < 0.001**
No-Severe	** < 0.001**
Mild-Moderate	**0.006**
Mild-Severe	**0.008**

*In post-hoc with Bonferroni correction, indicated that there is a statistical difference between the groups in terms of symptoms and emotions

## Discussion

There are no agreed-upon guidelines for the diagnosis and management of uremic pruritus, which has resulted in underdiagnosis of the condition and generally subpar patient care. According to reports, the prevalence varies greatly between nations as well as between centres within one nation. Pathogenesis is linked to a number of risk factors, many of which are not fully understood. The majority of the time, pruritus is persistent and recurrent, bilaterally symmetrical, affects the trunk, back, and limbs, has no primary skin lesions, and is worse at night. The patient’s QOL is significantly impacted by CKD-aP, which has a negative impact on their sleep and social functioning. There is no one treatment that works for all cases of uremic pruritus because there are numerous contributing factors, and these factors differ greatly between patients [1, 2, 7, 8].

In this study of 120 patients analysed, we found the prevalence of uremic pruritus to be 55.8% (n = 67) in our maintenance hemodialysis patients which is similar to the Indian study by Kaur et al. [10] in 2019 wherein a total of 164 patients were analysed and they found to have a prevalence of 53.7% and is also similar to a Chinese study by Zhao et al. [11] study, which found 60 cases of uremic pruritus among 148 eligible patients (40.54%). According to some older series, the prevalence of uremic pruritus among patients receiving hemodialysis ranges from 22–84% to as high as 90% [12–18].

The average age in this study was 56.5 ± 11.3 years, 55 (82%) were males, 16 (23.8%) had diabetic kidney disease, 64 were non-smokers and 4 patients were seropositive. This was similar to a study by Kaur et al. [10] wherein the average age of the cohort was 52 ± 17 years, and it included 95 (57.9%) males and 69 (42.1%) females, diabetic kidney disease (n = 70, 42.7%) was the commonest cause for end-stage kidney disease, 8 were active smokers and 13 were seropositive. Our study is also similar to the Chinese trial by Zhao et al. [11] wherein among 148 eligible subjects 89 were males (60.1%) and 59 females (39.9%) with diabetes seen in 35 (23.6%) patients.

In our study among 67 patients, 49/67 (40.8%) had a mild itch, 13/67 (19.4%) had a moderate itch and 5/67 (4.1%) had a severe itch. This was slightly deviant from other studies where a majority of the studies have shown moderate itch to be the commonest such as Singh et al. [19] wherein 23.3% had “mild itch”, 53.4% had “moderate itch” and 23.3% of them had “severe itch”; Zhao et al. [11] showed 22 (14.8%) cases of “mild itch”, 30 (20.2%) cases of “moderate itch” (20.27%), and 8 (5.4%) cases of “severe itch” (5.41%); Kaur et al. [10] displayed mild itch in 28 patients (31.8%), moderate itch in 40 patients (45.5%), and severe itch in 20 patients (22.7%).

In our study, there was no significant association between clinical characteristics and uremic pruritus which is similar to several other studies [20–22] wherein no statistically prominent association was noted with factors such as age, sex, etiology of uremia, smoking, and seropositive status. In this study, there was also no significant association observed between dialysis-related factors such as vintage, duration, frequency, and adequacy of dialysis as well as laboratory factors traditionally involved such as anaemia, hypoalbuminemia, hypocalcemia, hyperphosphatemia, and high levels of intact PTH which is also observed in several other studies [20–22] wherein no positive association was seen with low hemoglobin, low serum albumin, low calcium, high phosphorus, and high levels of PTH in those with pruritus among patients of end-stage kidney disease.

In our study with increasing severity of pruritus, the SKINDEX-10 showed worsening symptom-related, emotional, and functional quality of life parameters. Similarly, Itch-MOS revealed that they had poorer sleep quality and quantity, as well as worsening renal itch. Numerous other studies have proven a connection between pruritus and poorer quality of life, insomnia, depression, an independent predictor of mortality, and other detrimental patient outcomes. However, very few studies have systematically correlated the severity of pruritus with various parameters, as ours has. Additionally, according to Lopes et al. [23], patients with severe pruritus had a 25% lower quality of life-related to the burden of kidney disease, primarily as a result of sleep disturbances, depressive symptoms, and dry skin. Mathur et al. [24] found a statistically significant link between the severity of pruritus and “HR-QOL”, particularly in terms of mood, social connections, and sleep. They also found that a 20% change in itching intensity over time was associated with clinically significant changes in HR-QOL. The largest study to look into HR-QOL was carried out by DOPPS [5], utilizing general scales ["36-item short-form health survey (SF-36) and 12-item short-form health survey (SF-12),"] and proved that those with more itching had poorer HR-QOL. Similar findings were also made by Kosmadakis et al. [25] and Tessari et al. [26]

## Limitations

- Single-center, short-term observational study.
- Plethora of other factors that influence pruritus; testing for all of them was not practical.
- Other factors affecting the quality of sleep such as the presence of OSA (obstructive sleep apnea), use of sedatives, or anxiolytics were not analysed
- Therapeutic interventions were purposefully excluded because compliance could not be determined due to limited subjective benefits and multi-therapy.

## Conclusion

Uremic Pruritus was found in 55.83% of patients, with mild pruritus being the commonest. There was no significant relationship between uremic pruritus and clinical characteristics, dialysis-related factors, or biochemical parameters that were traditionally known to influence. As the severity of the pruritus worsened, these patients had the worst HR-QOL, sleep quality, and sleep hours.
